# Dengue

**Published:** 1829

**Authors:** 

**Affiliations:** Savannah


33. *Dengue.* In the same Journal, Dr. Daniell, of Savannah, has published the results of his observations on this singular and novel malady, of which we have already taken an extended notice. Desirous of putting our readers in possession of all the important facts touching this strange epidemic, we shall present them with such paragraphs of the Doctor's short paper, as will present his pathologic views and the practice which he pursued.

The Dengue is certainly an exanthematic fever. At an early period, an eruption invariably appears in some part of the body, unless prevented by active purging, bleeding, or some other injudicious prescription. Upon fixing and maintaining this eruption on the surface, and allowing it gradually to pass off by perspiration, depends the radical cure of the disease.

As just stated, my great object in treating Dengue was to fix the disease upon the surface. To this end, upon visiting a patient laboring under it, and especially in the first stages of the disease, I directed a mustard plaster to be placed on the abdomen to inflame the skin. If the eruption had previously existed, this increased it, or in its absence produced it. In some cases additional plasters of the same article were placed on the extremities. Whenever the eruptions were fully established on the surface, the pains of the different parts of the frame were removed.

If circumstances indicated their use, castor oil, or some mild laxative was given, to obviate or remove costiveness.

The eruptions having been fixed on the surface, serpentaria, and bark or quinine were given, until the fever subsided, which was commonly from two to four or five days. After that, the serpentaria was given alone for six or eight days, by which a constant and gentle moisture was maintained on the surface. During this latter period, animal food, porter, or wine, were given the patient in small quantities. Under the above briefly recited treatment, the eruptions gradually passed off, (usually before the fever,) and the patient was restored to health, without being subjected to the usual consequences of the disease, as pains in the limbs, swellings and stiffness of the joints, which appeared to run on for months, and afterwards might be renewed by any slight exposure.

When the skin has been inflamed, the eruptions increase, and in many instances run into each other, producing the appearance of a deep blush over the whole face, neck, and breast. When a moisture has been produced to some extent, there is an abatement in the deep colour of the eruption. This eruption, as before remarked, gradually subsides.

Where the eruption has been regarded as an accidental symptom, and allowed to disappear, or where it has been repelled or prevented from appearing, by the use of purges, the lancet, antimonials, &c. almost every part of the system is liable to be attacked. Several cases of mania, consequent upon repelled eruptions, occurred within my observation. Dr. Foskman informed me of another, in his own practice. In a lady it produced an inability to retain her urine. The most singular consequence which I witnessed, however, as resulting from repelled eruptions, was a case of general tetanus, which terminated fatally.—p. 292.

